# Hospital Meetings, &c.

**Published:** 1899-03-04

**Authors:** 


### HOSPITAL MEETINGS, &C.

### CHARING CROSS HOSPITAL.

The Bishop of London (Dr. Mandell Creighton) presided at the seventy-eighth annual General Court of Governors of the Charing Cross Hospital on Wednesday, 22nd ult. The report for 1898 gave the number of patients treated during the year as 21,771, of whom 2,155 were in-patients, 10,466 out-patients, and 9,150 casualties; and an analysis of the accounts shows that the cost of each in-patient was £5 14s., and the cost per bed £70 6s. The Council express gratification that the financial position reached in 1897 was maintained during 1898. Since the special appeal was started two years ago, not only have all the expenses of maintenance been met, but the debt of £17,000 has been paid off, £16,144 has been expended in the purchase of the freehold of the houses immediately needed for the improvements and the lease of 17, Chandos Street; and, in addition, £7,500 has been invested from special gifts for the endowment of beds. The ordinary income for the year as shown in the accounts was £9,024, in addition to legacies of some £10,000, and the special appeal fund amounting to £5,000, in raising which £768 was expended; £1,156 was received on the convalescent home account, and £1,453 expended. The ordinary expenditure is shown as £14,264, and the extraordinary expenditure as £605.

In moving the adoption of the report and accounts the Chairman said that though he had no detailed knowledge of the working of the institution, he was present two years ago when a special appeal for funds was made, and he remembered that at that time a certain gloom was prevalent, but he was glad to learn from the report that this had been dissipated. The public had a right to know, and had been demanding to know, if the charities it was asked to support were really deserving of that support. He was, therefore, glad to find that the appointment of an inquiry officer had afforded satisfactory information on that point. In only 52 cases out of over 6,000 was it found that the applicants were not fit subjects for charitable relief. That showed that the great mass of the people applying for help were deserving of it. This was most satisfactory, and the presence of that officer was a guarantee that that would continue to be the case. Several other points in the management of the hospital required attention. Medical science was continually making greater demands, and he was glad to learn that a new department had been formed for the Röntgen apparatus, with a medical officer in charge. But, good as they were in many ways, they always wanted to be doing better. They desired to be showing progress, and surely nothing ought to show more progress than charitable work. They ought never to be satisfied with their progress and their aspirations till they could say to the poor and needy, "This is the very best the community, as a whole, can possibly supply." In the wards they had done this; but other departments were not what they should be. The out-patients' room was dimly lighted and provided little accommodation, while the medical staff and nurses were badly housed. Within the recollection of all of them nursing had come to be recognised as of much greater importance than was formerly the case, but there had been no adequate increase of accommodation for the staff. Great demands were made on their strength and cheerfulness, but he was afraid their comfort had not hitherto been greatly considered. But the improvements now contemplated would provide for the out-patients, for the nurses, and the resident physicians, besides wards for special cases. It was computed that £50,000 would be required, and the council had wisely determined not to begin operations till they saw their way to at least half that sum. He felt sure the public would respond to the appeal now made, and enable them to complete the hospital on the site which they had at last acquired after so many years of waiting and so many disappointments.

Mr. J. Swayne Pearce seconded the motion, which was carried unanimously.

In supporting a vote of thanks to the Bishop for presiding, Prebendary Kitto said the British public had done enough already in response to the appeal to show that it did not want them to give up their task, and now they meant to keep hammering away until the British public succumbed and gave them all that was wanted.

### ROYAL FREE HOSPITAL.

The seventy-first annual meeting of the Royal Free Hospital, Gray's Inn Road, was held on Wednesday, 22nd ult., Mr. Justice Bruce, the chairman of the committee of management, presiding. The accounts presented gave the ordinary income as £9,775, and legacies as £4,063, a total income (exclusive of building improvement fund) of £13,838. The ordinary expenditure was £12,313, plus extraordinary repairs £386, a total expenditure of £12,699. In their report the committee express regret that though new subscribers were obtained, the year shows a falling off in the amount received under that head, resulting not only from the loss by death of liberal supporters, but also from the withdrawal of subscriptions by several firms whose businesses have been converted into companies. The committee consider the time has come when a commencement should be made in carrying out the more urgently needed of the improvements necessary to bring the hospital up to the standard of modern requirements, viz., alterations and additions to the ward lavatories and bathrooms; and better accommodation for the nursing staff by providing separate bed-rooms for 27 sisters and senior nurses, an enlarged sitting-room, additional bath-rooms, &c. All these improvements were strongly recommended by the visitors who inspected the hospital on behalf of the Prince of Wales's Fund. The total cost of the works is estimated at £8,500, towards which here is so far available from legacies and donations a sum of £2,870, and a special appeal is made for contributions towards this fund so that the improvements may be carried out during the present year. The medical reports show that the number of patients treated during the past year was 38,688, as compared with 37,280 in 1897. Of these the in-patients numbered 2,151 and the casualties 16,139. There are 170 beds available, and the average weekly cost per bed during 1898 was £1 7s. 7d. The work of Miss Stewart, the almoner, has been continued, and the value of systematic inquiry into the circumstances of persons applying for treatment is becoming more recognised every year, the knowledge that inquiry is held acting as a wholesome check against abuse of charity. During the year 70 persons were refused treatment beyond that first received, on the ground that they were able to afford to pay for private advice.

The Chairman, in moving the adoption of the report, referred to the deaths during the year of Lord Lathom, Sir Stuart Knill, and Mr. Alfred Cock, Q.C., all valued friends of the hospital, to whose memory he paid a warm tribute. Miss Mabel Webb also, the curator of their museum, had died very suddenly, to their great regret. As a memorial of her zealous work it had been resolved to carry out certain structural alterations and additions to the museum which Miss Webb had herself proposed. Continuing, the Chairman said that the great topic dealt with in the report was, of course, the question of the improved accommodation for the staff. There was no doubt as to the necessity for securing the best nurses if they were to get good results, and if they wished to retain these nurses they must provide them with proper accommodation, or, naturally, they would go elsewhere.

Mr. Chas. Burt, the chairman of the Weekly Board, seconded the motion, and strongly emphasised the need for the contemplated improvements. He had, he said, as a member of the Prince of Wales's Fund Visiting Committee, recently had an opportunity of seeing what other hospitals had done in this matter, and he came back convinced that they could delay no longer. Forty years ago cubicles were considered good and sufficient for nurses, but few institutions were now content with such arrangements. When they considered the need for patience and cheerfulness on the part of the nurses, and how little change they had, he felt sure the committee would not appeal in vain for the necessary funds. They wanted £6,000 out of the estimated £8,500 at once; and they were emboldened to press for it since comparison showed that no hospital was more economically managed than theirs. What money they had they were using well. He appealed not only to the general public, but to their students as well, and to those who had passed through the medical school, to do their best to help.

Dr. West heartily endorsed what had been said as to the absolute necessity for improved accommodation for the doctors' right hands—the nurses—and the resolution was adopted unanimously.

Earl Stanhope was appointed as a vice-president of the hospital, and Mrs. Garrett Anderson, as usual, proposed the vote of thanks to the Management Committee, which was cordially carried. Votes of thanks to the treasurer, the medical staff, the Ladies' Committee, and to the chairman having been passed, the meeting came to a close with a suggestion from Mr. Rundall that the vestry should be approached on the subject of the rates. Mr. Burt said the matter was under the consideration of the General Hospital Committee for London with a view to making a united effort in the matter.

### KING'S COLLEGE HOSPITAL.

The sixtieth annual meeting of King's College Hospital was held on Thursday, 23rd ult., Mr. G. W. Bell in the chair. The Committee of Management in their report congratulated the governors on the results of the year's work. The total expenditure for 1898 was £18,458—£207 less than the previous year. Of this, £523 was spent on the convalescent home. The total ordinary income was £12,392, with legacies of £5,901. The number of in-patients was 2,561—slightly above the average; in the out-patient department the new cases were 23,353, of which 11,535 were accidents, 577 being those of poor women attended during confinement at their own homes. Attention was called to the heavy, but absolutely necessary, work of reflooring the wards, towards which £475 had been received from the Prince of Wales's Fund; at least £3,000 will be required to complete the work In succession to Mr. Richard T[illegible], who retired after 13 years of office, Mr. C. Awdrey was in February last unanimously elected treasurer of the hospital. During the year three wards have been fitted for electric lighting at the cost of generous friends, and the committee has reason to hope that three more will be lighted this year. The report and accounts were adopted, and votes of thanks to the committee, officers, and staff concluded the proceedings.

### ROYAL NATIONAL HOSPITAL FOR CONSUMPTION.

The annual general meeting of the Governors of the Royal National Hospital for Consumption and Diseases of the Chest (on the separate principle), Ventnor, was held on Thursday at the London office, 34, Craven Street, Charing Cross, under the presidency of Sir Richard E. Webster, Q.C., M.P. The thirtieth annual report which the board of management submitted for adoption stated that during the year 1898 840 patients had been treated, the duration of their stay at the homes varying from a week to 144 days, whilst at the end of the year 129 medically accepted applicants were waiting admission. During the present year 21 additional beds will come into use by the opening of the eleventh block of houses. The medical report showed that of the 840 patients 711 completed their term during the year, 147 (20·67 per cent.) were restored, 418 (58 79 per cent.) were much improved, that 129 (18 28 per cent.) were unimproved or worse, and that 17 (2 39 per cent.) died, the death-rate calculated from the 840 patients treated being 2 per cent. Funds for furnishing the new block are needed. The ordinary receipts amounted to £11,401, plus legacies £10,970—a total of £22 371. The ordinary expenditure was £11,594 and the extraordinary expenditure on building and alterations amounted to £7,373, together £18,967 £4,340 was invested, and £1,000 having been borrowed from the bankers, and £395 brought forward, the cash balance at December 31st last was £459. The outstanding liabilities (beyond current maintenance expenses) amount to £6,550.

The Chairman, in moving the adoption of the report, referred to the invaluable work which was carried on by the hospital and pointed out that by means of the successful treatment afforded valuable lives had been saved and many patients had added many useful years to their lives by a sojourn at the institution. They had experienced a very successful year. It had amused many of them connected with the hospital to note that the open air treatment was being regarded as a new thing. But it was no new thing; its value had been recognised in Ventnor from the first, and it was one great advantage of their buildings that from their situation they could supply patients with plenty of fresh air and sunshine, the great advantage of which they had urged on all occasions. Sir Richard also briefly alluded to the additions to the institution, the improvement in the water supply, and the installation of the electric light during the past year, and added that, of course, these had incurred an increased annual outlay. They were encouraged in their work in spite of the increased number of applications, on which, however, they seemed not to make an impression, in spite of continual additions to their houses. He concluded with an earnest appeal for aid in the work of battling with this terrible scourge. The more effective their hospital the greater were the demands on their funds.

Dr. J. Sinclair Coghill, the physician to the hospital, dealt with the medical report, and read statistics showing that the results obtained by the English methods of treatment such as were in force at Ventnor compared favourably with those at Continental sanatoria. The report was adopted.

The Treasurer (Mr. P B. Burgoyne), who was re-elected, called attention to the outstanding liabilities, and stated that they had, since the report was drawn up, been obliged to sell £2 000 of their invested capital stock.

### THE CANCER HOSPITAL, BROMPTON, LONDON, S.W.

The forty-eighth annual meeting of the Governors of this charity was held in the boardroom of the hospital, Brompton, on Wednesday, the 22nd ult. Sir George S. Measom, J.P., occupied the chair, and amongst those present were Major-General T. M. Bailie, Mr. G. L. Beeforth, J.P., Mr. D. J. Chattell, Major Robert Gordon-Gilmour, Mr. Edward Herdman, Mr. William Hooper, Mr. P. O. Margetson, Lieut.-Colonel T. R. Parr, Mr. Archibald Sturrock, Mr. F. R. Wegg-Prosser, Lady Measom, Lady Helen Boyle, Mrs. and Miss Liberty, Dr. W. A. Satchell, J.P., Dr. Snow, Dr. Purcell, Dr. Bourns, Mr. Ryall, Mr. Willard, the Rev. F. W. A. Wilkinson, Miss Rogers (matron), Mr. F. W. Howell (secretary), &c. From the report of the committee it appeared that during the past year 2,477 new patients were received, 835 being in and 1,642 out patients, whilst the total number of attendances of out patients was 13,803. Much regret was expressed at the untimely deaths of Mr. W. H. Hughes (secretary) and Mr. Edward Cotterell, an able and valued surgeon of the hospital for the past six years. The committee regret that there had been a considerable falling off in donations and annual subscriptions during the past year, and desire to impress upon their friends the necessity of increasing the donations and annual subscriptions, which are practically the main support of the hospital. During the year the question of the appointment of a successor to the position of secretary had received full consideration, and out of about 300 applications for the office Mr. F. W. Howell, of the York County Hospital, was elected to the office of secretary, and Mr. C. Jarman appointed assistant-secretary of the hospital, in appreciation of his services for 26 years. The Samaritan Funds had proved of great advantage to the patients in assisting them to go to their own homes and to convalescent homes. The report and balance sheet were adopted, and the usual votes of thanks accorded, after which the proceedings terminated.

### EAST LONDON HOSPITAL FOR CHILDREN.

Viscount Falkland, the vice-chairman, presided at the annual Court of Governors of the East London Hospital for Children, Shadwell, on Thursday, 23rd ult. The accounts presented showed that the ordinary receipts for the past year were £6 829 and the ordinary expenditure £7,953.

In moving the adoption of the report, the Chairman remarked that in the year just ended the institution completed the thirtieth year of its existence. During that time free treatment had been afforded to 26,236 in-patients and 476,284 out-patients, a total of close upon half a million, mostly children. During the past year the number of in-patients had fallen to 1,512, as compared with 1,616 in 1897; but on an average these had remained two days longer, the daily average of occupied beds in 1898 being 88·12, the period of residence of each patient being 21 27 days. Only £1 775 was received as legacies during 1898, as against £5 493 in 1897, and there was also a small falling off. One thousand pounds was received for the endowment of a cot, and a donation of £250 from the Prince of Wales's Fund towards a new operating theatre, which the member of the council who inspected the hospital reported as most necessary. The convalescent home at Bognor was now paid for, but £1,4(0 was required for furnishing and to meet the cost of a small isolation block—the need for which had already made itself felt—and a laundry. Since the end of the year a gentleman had given £500 towards this, and it was hoped the remaining £400 would be specially subscribed. The cost of maintenance of the home would be at least £600 a year, and efforts were being made to obtain special subscriptions and donations towards this. With regard to the new operating theatre, estimates had been received from four different firms, the highest being £1,835 and the lowest £1,766—all far in excess of what had been expected. It was thought, however, that a reduction of some £250 might be effected by modification of some of the items, thus leaving £1,266 still to be collected. The sale of the old freehold premises on which the hospital once stood had been arranged for the sum of £725, which was considered a very good price. A whooping-cough ward was badly wanted. It was strange that in a children's hospital they should have to refuse admittance to cases of the disease which stood second among those most fatal to the young. Negotiations were, however, pending for the acquisition of a plot of ground adjoining the hospital on which it was proposed to build the laundry, post mortem room, and mortuary, the space now occupied by these offices being thus made available for a special whooping cough ward. The report having been adopted, Mr. Bernard Ford moved that one general court per annum be held in the evening, to allow of the attendance of working-men governors, representatives of societies, but after some discussion it was decided that it would be better to give notice that the motion would be brought forward at the next meeting, and this was accordingly done.

### QUEEN CHARLOTTE'S LYING-IN HOSPITAL.

THE annual meeting of Queen Charlotte's Lying-in Hospital, Marylebone Road, W., was held on February 27th at half-past four, the Earl of HARDWICKE in the chair. There were present: The Rev. Sir Talbot Baker, Bart., Captain Gilpin, Major McMahon, Mr. F. W. Hunt, Dr. Griffith, and Mr. R. Martin. The report, which was introduced by a few remarks from the Chairman, was taken as read, and carried unanimously. It stated that the year had been a prosperous one, the charity having been well maintained, and the patients benefiting by it reached the large number of 2,182. There was no case of septicæmia, and only three deaths occurred. The income showed an increase under nearly every head, the total amount being £3,501. The expenditure was only £8 more than last year, and amounted to £4,123. The visiting committee of the Prince of Wales' Hospital Fund expressed their approval of the arrangements under which the hospital was conducted, and made a grant of £300, as well as a special grant of £50 to the Convalescent Fund; £150 was received from the Hospital Sunday Fund, and £96 5s. from the Hospital Saturday Fund. £3,059 was received in legacies. The midwifery training school maintained its high reputation, and applications for admission are received in growing numbers; candidates from Canada, India, and Denmark. The convalescent home proves a valuable adjunct to the hospital. A hundred and eleven patients, making a stay averaging four weeks each, visited it last year at a cost of £429. Some additional expense has been incurred by putting the houses in thorough repair. The Earl of Hardwicke has accepted the chairmanship of the Committee of Management. About £8,755 has been already subscribed towards the enlargement of the hospital and new nurses' home, but a further sum of £7,000 is needed to meet the liabilities on these scores. The new home for the nurses will be opened in the summer, and the committee are most anxious that the buildings may be then free from debt.

### GREAT NORTHERN CENTRAL HOSPITAL.

SIR JOHN DICKSON-POYNDER, M.P., presided at the annual general meeting of the Great Northern Central Hospital on Friday last (24th ult.).

THE CHAIRMAN, in proposing the acceptance of the report of the Committee of Management, referred in most appreciative terms to the services rendered to the institution by their late chairman, Mr. Murdoch, whose death accounted for the speaker's occupancy of that position. They all, he knew, lamented the sad event, and the irreparable loss, which would be felt not only in philanthropic, but in political, in social, and in commercial circles as well. Another great loss the hospital had sustained during the past year was that of their obstetric physician, Dr. Leonard Remfry; while owing to the many calls upon him, Mr. Lockwood, one of their surgeons, had felt compelled to resign his position with them, a severance they greatly regretted also. Taking the accounts on their broad lines, they indicated a steady growth of prosperity during the year. There had been an increase in the annual subscriptions, bringing that most important source of revenue up to £2,016, while ordinary donations amounted to £3,100. The ordinary income for the year was £8,090, which, with £7,550 from legacies, made an income available for maintenance and management of £15,640, and with £2,835 in donations to the Ladies' Association Endowment Fund and £210 to the General Endowment Fund account, made a total revenue of £18,685—a higher figure than they had ever before reached. The ordinary expenditure amounted to £10,183, £247 being spent on repairs and furniture, while £3,012 was invested, leaving a balance to account of Surplus General Fund of £5,242. This year they had made a new departure, it having been decided that half the money raised by the Ladies' Association should be devoted to the Endowment Fund and half for current maintenance expenses. £813 of the donations mentioned was received from the Islington Diamond Jubilee Fund, the contributors stipulating that it should be utilised by at once opening one of the unused wards. This was accordingly done, the number of beds available being raised on October 1st from 107 to 134. A large number of additional cases were thus enabled to be admitted, and from the report it appeared that the number of in-patients had risen to 1,701 as compared with 1,527 in 1897. The out-patients rose in number to 28,160, an increase of 2,699. These increases were not due to any relaxation of the inquiry system, but to the greatly extending population of North London and to the fact that many more patients now come from the locality instead of going to hospitals in the centre of London. The subscription of £750 from the Prince of Wales's Fund was also commented on. A committee from the fund inspected the hospital in the summer, and expressed themselves very pleased with its arrangements, but the committee were hardly prepared for so substantial a grant, nor for the most acceptable announcement which accompanied it, namely, that the grant would be an annual one, subject to fifteen of the unoccupied beds being opened for use. In accordance with this, immediate steps were being taken to open Ward IV.—a step which would entail an estimated added cost of about £850 per annum. An increase of five in the nursing staff, two extra servants, and a porter would be needed, but no addition to the management staff would be necessary. The Chairman pointed out that all the wards of the hospital being now open and more beds in use, they would get a larger amount *pro rata* from the Hospital Saturday and Sunday Funds. In a short time they would have 159 beds available, and their expenditure when in full working order would be about £11,000. The average number of beds occupied during the year was 107·8, and the cost per bed worked out at £71 8s. 9d., a reduction of £3 18s. 11d. on that of 1897. The management expenses were now

14½ per cent., as compared with 18 per cent., and they hoped to show a still further reduction in both these figures. They hoped soon to have the electric light, an operating theatre for out-patients, a laundry, and several other improvements, and thus maintain their high place in public estimation.

Mr. Austin seconded the resolution, and warmly welcomed the new chairman in words which were received with hearty applause.

The report was adopted without discussion, after which ten members of the committee of management were re-elected, as were the hon. treasurer and hon. secretary.

The chairman was elected a trustee of the institution, after which a motion was proposed by Mr. W. T. Rose to reduce the subscription required from firms and congregations to entitle them to a representative on the governing council, from 50 guineas to 30 guineas. After some discussion this was negatived by a large majority, and votes of thanks brought the proceedings to a close.

### THE ROYAL PORTSMOUTH HOSPITAL.—OPENING OF NEW WARDS.

On Monday T.R.H. the Duke and Duchess of York paid their promised visit to Portsmouth to open the new wards of the Royal Portsmouth Hospital, a Diamond Jubilee Commemoration, erected at a cost of about £16,000. The buildings are only just finished and are not yet furnished throughout; only the upper ward in block A was resplendent in its completed glory. The opening ceremony took place in the lower ward of this block, where a platform was erected and space found for some of the many guests who thronged the hospital and its precincts. The chairman of the hospital, Sir W. D. King, the vice-chairman, the Rev. G. P. Grant, the Mayor, members of the medical staff, and Miss Boulton, the matron, received the Duke and Duchess at the main entrance, and conducted them, after various presentations had been made, to Ward I. Prayers were here said by the Chaplain, the Rev. C. Crowley, and speeches followed from the Chairman, Lord Northbrook, Sir John Baker, and Dr. Ward Cousins. In reply, the Duke of York declared the wards opened, naming the two for women "Victoria" and "Duchess of York," and the male wards "Albert" and "Connaught." Their Royal Highnesses then inspected the furnished ward upstairs and glanced into the others, winding up with a visit to the accident ward in the old building to see one of the survivors of the collier "Arno," Fireman William Johnson, whose leg had been amputated in consequence of his severe injuries. Tea was served in the nurses' dining-room, and soon after five o'clock the Royal visitors departed after inspecting the volunteers' guard of honour drawn up before the entrance. The completed ward in block A looked very charming with its spotless new fittings, and decked with plants in honour of the occasion. The furniture is all of polished Kauri pine, the walls coloured pale yellow, with a dado of dark brown, and red screens supplied a cheerful note of colour. The ward is heated with two Pridgin Teale stoves, double grated, fitted with grey marble tops and golden brown tiles. Between the stoves are large tables, 9 ft. by 6 ft., with capacious cupboards and drawers. The beds are after the pattern of those at the Birmingham General Hospital; the lockers are marble-topped, and provided with cupboard and drawer, and brass towel rail. There are easy chairs and wheeled chairs; electric lights in standards (arranged in such a way that they may be conveniently shaded at night), and round the walls, with also a switch beside each bed for a handlamp. The lavatories are walled with white glazed bricks, and an excellent plan has been adopted in the addition of a small ventilated cupboard, built in the wall, and closed with an iron door, to receive anything saved for inspection. The finished ward gives promise that the whole building, when completed, will be quite a model hospital, and we wish all concerned a speedy end to the present stage of transition, always so trying an experience, and the hastening of the joyful day which will see the new wards occupied.

